# TNF-*α* Increase in a Cohort of Depressive Patients

**DOI:** 10.1155/2021/8897421

**Published:** 2021-03-15

**Authors:** Adrian Groh, Kirsten Jahn, Marc Walter, Johannes Heck, Ralf Lichtinghagen, Eva Janke, Schulze Westhoff M. L, Maximillian Deest, Helge Frieling, Stefan Bleich, Kai G. Kahl, Annemarie Heberlein

**Affiliations:** ^1^Department of Psychiatry, Social Psychiatry and Psychotherapy, Hannover Medical School, Hannover, Germany; ^2^Department of Psychiatry (UPK), University of Basel, Basel, Switzerland; ^3^Institute for Clinical Pharmacology, Hannover Medical School, Hannover, Germany; ^4^Institute of Clinical Chemistry, Hannover Medical School, Hannover, Hannover, Germany

## Abstract

**Background:**

The model of neuroinflammation has been proposed as a possible explanation of depression. Investigations of serum levels of tumor necrosis factor-*α* (TNF-*α*) in depressed patients have previously shown contradictory results of increased and decreased levels of TNF-*α* during the treatment of depression.

**Methods:**

We compared the serum levels of TNF-*α* in two cohorts of patients suffering from depression (ICD-10 criteria): one cohort from a psychotherapeutic unit (*n* = 18), where patients were treated with Cognitive Behavioral Analysis System of Psychotherapy (CBASP), and the other cohort from a psychiatric day care unit (*n* = 16). Both cohorts were investigated at the beginning and at the end of treatment. The intensity of depression was measured by means of the Beck Depression Inventory, 2^nd^ edition (BDI-II) at both time points.

**Results:**

We observed a statistically significant increase of TNF-*α* in the psychotherapeutic unit at time point 2 compared to time point 1 (*T* = −14.71, *p* < 0.001), but not in the psychiatric day care unit. In both cohorts, BDI-II scores at time point 2 were significantly decreased compared to time point 1 (psychiatric day care unit: *T* = 3.32, *p* = 0.005; psychotherapeutic unit: *T* = 6.22, *p* < 0.001). There was a significant correlation in the psychotherapeutic unit at time point 2 (*r* = −0.682, *p* = 0.02).

**Conclusion:**

As TNF-*α* was increased at time point 2 in the psychotherapeutic unit but not in patients of the psychiatric day care unit, we propose the different durations of pretreatments in both cohorts and the associated processes of neuroinflammation as a possible explanation for our results. The lack of information about the time course of TNF-*α* in depression could in general explain the huge variety of TNF-*α* levels in different cohorts of depressed patients reported in the literature.

## 1. Introduction

Depression is one of the most prevalent psychiatric diseases. The World Health Organization (WHO) estimates that 322 million people worldwide suffer from depressive disorders [1]. Depressive disorders are characterized by sadness, low mood, lack of energy, and the inability to enjoy life [2]. Depression is diagnosed by clinical examination and diagnostic identification according to the International Statistical Classification of Diseases and Related Health Problems, 10^th^ Revision (ICD-10) [3] or the Diagnostic and Statistical Manual of Mental Disorders, 5^th^ Edition (DSM-5) [4] assisted by psychometric tests such as the Beck Depression Inventory (2^nd^ edition, BDI-II) [5] or Hamilton Depression Rating Scale (HDRS) [6].

Therapeutic success is usually evaluated by clinical improvement and a decrease of scores on depression scales. Moreover, especially in recent years, putative biomarkers for depression have been discussed [7]. From the hypothalamic-pituitary-adrenal axis (HPA) hormone cortisol, which is perhaps the most intensely studied biomarker for depression [8], to the neurotrophic (growth) factors [9], which have more recently gained scientific attention, there is a plethora of research [10]. This research also includes parameters supporting the inflammatory hypothesis of depression [11]: myeloperoxidase, inducible nitric oxide synthase, pro- and anti-inflammatory cytokines, and the phenomenon of oxidative stress may lead to a deficit in serotonin and melatonin via the kynurenine pathway, which is considered as one of the main reasons for depression. In a meta-analysis from 2015, Strawbridge et al. hypothesized that prospectively determined treatment resistance may be associated with increased inflammation [12]: consistently, the data showed a decrease in tumor necrosis factor-*α* (TNF-*α*) serum levels over time in treatment-responsive but not in treatment-resistant patients. TNF-*α* is known as a cytokine, which is, besides other functions, responsible for the acute phase reaction of systemic inflammations.

According to national and international guidelines, psychopharmacotherapy (particularly antidepressants), psychotherapy, and equipment-based treatments such as light therapy and electroconvulsive therapy represent the cornerstones of the treatment for depression [13–15].

With regard to the model of neuroinflammation, TNF-*α* has also been proposed as a possible target for antidepressant treatments, for example, via treatment with TNF-*α* antagonists such as adalimumab, infliximab, or etanercept [16–19].

The Cognitive Behavioral Analysis System of Psychotherapy (CBASP) was developed by J. McCollough and is explicitly designed as a treatment of chronic depression [20]. CBASP is the subject of significant research and is very well validated [21].

To the best of our knowledge, this is the first study to investigate the effects of CBASP on TNF-*α* levels in depressed patients.

## 2. Materials and Methods

This trial was approved by the ethics committee of Hannover Medical School. The research was conducted in accordance with the World Medical Association Declaration of Helsinki. The participants received verbal and written information and gave their written informed consent before inclusion.

Here, we present data from two different cohorts: one cohort was recruited in a psychiatric day care unit (*n* = 18) and the other cohort in an inpatient psychotherapeutic unit (*n* = 16) with a focus on depression (see Table 1 for demographic data). In both cohorts, the subjects fulfilled the diagnostic criteria (according to ICD-10) of a moderate depressive episode (F32.1) or recurrent depressive disorder, current episode moderate (F33.1), or more severe forms of depression (F32.2, resp., F33.2).

The patients from the psychiatric day care unit received a general psychiatric treatment with psychoeducation, supportive talks, etc. Patients on the psychotherapeutic units were treated with either cognitive behavioral therapy (CBT) [22] or with the Cognitive Behavioral Analysis System of Psychotherapy (CBASP) depending on their type of depression. CBT is seen as a standard psychotherapeutic treatment for patients with primarily episodic forms of depressive disorders. CBASP has been developed as a treatment for chronic major depression and is foremost a behavioral analytic therapy, targeting interpersonal consequences of behavior [23].

The following comorbidities represented exclusion criteria: posttraumatic stress disorders, severe organic diseases (e.g., oncologic diseases), psychotic or bipolar disorders, and alcohol and/or substance abuse.

All study participants completed the treatment and were electively discharged from hospital.

There were two time points of blood withdrawal and collection of psychometric data: at the beginning (T1) and at the end (T2) of treatment Psychometric data were obtained using the BDI-II. The BDI-II is a multiple-choice self-report inventory, measuring the severity of depression via21 items, each item ranging from 0 to 3 points [5].

### 2.1. Laboratory Analyses

#### 2.1.1. TNF-*α* Measurement

The quantitative measurement of TNF-*α* was performed using a solid-phase chemiluminescent immunometric assay (Siemens Healthineers, Erlangen, Germany). Samples were measured undiluted according to the manufacturer's instructions on an Immulite 1000 analyzer. The analytical sensitivity was at 1.7 pg/ml, and the measuring range was up to 1000 pg/ml; intra-assay and interassay precision was <5% and<7%, respectively.

### 2.2. Statistical Analyses

All statistical analyses were performed using the Statistical Package for the Social Sciences (SPSS™) for Windows 26.0 (SPSS Inc., Chicago, IL) and Graph Pad Prism™ 8.0 (Graph Pad Software Inc., San Diego, CA).

The hypothesis of a normal distribution of TNF-*α* levels and BDI-II scores was accepted by means of the Kolmogorov–Smirnov test.

Correlations of the TNF-*α* levels and BDI-II scores were calculated by Pearson's correlation coefficient. Age, gender, body mass index (BMI), and treatment with antidepressants and/or antipsychotics were included as potential confounders.

Group differences between psychiatric day care unit and psychotherapeutic unit patients were calculated using independent samples *t*-tests. Alterations of TNF-*α* levels and BDI-II scores pre- and posttreatment were assessed by the *t*-test for dependent samples.

Bonferroni correction was used to account for multiple comparisons.

The statistical tests were performed applying a significance level of *α* < 0.05.

## 3. Results

### 3.1. Psychiatric Day Care Unit

#### 3.1.1. TNF-*α* Serum Levels

**Figure 1:**
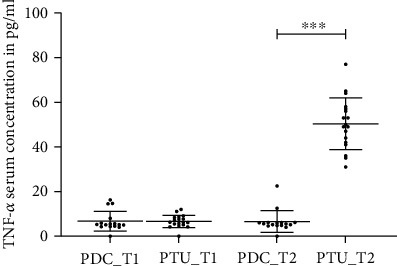
TNF-*α* serum level of the psychiatric day care unit cohort (PDC) and the psychotherapeutic unit cohort (PTU) at time points 1 (T1) and 2 (T2).

In the psychiatric day care unit cohort, the TNF-*α* levels were not statistically different at time point 1 (baseline) and time point 2 (data not shown, see Figure 1).

#### 3.1.2. BDI-II Scores

We observed a statistically significant reduction of BDI-II scores at time point 2 compared to time point 1 (*T* = 3.32, *p* = 0.01) in patients treated in the psychiatric day care unit (Table 1).

#### 3.1.3. Association between TNF-*α* Serum Levels and BDI-II Scores

There was no statistically significant correlation between TNF-*α* levels and BDI-II scores neither at time point 1 nor at time point 2 in patients treated in the psychiatric day care unit.

### 3.2. Psychotherapeutic Unit

#### 3.2.1. TNF-*α* Serum Levels

In the cohort treated with CBASP in the psychotherapeutic unit, we observed a statistically significant increase of TNF-*α* levels at time point 2 compared to time point 1 (*T* = −14.71, *p* < 0.002, Figure 1).

#### 3.2.2. BDI-II Scores

Furthermore, the psychotherapeutic unit cohort showed a statistically significant reduction of BDI-II scores at time point 2 compared to time point 1 (*T* = 6.22, *p* < 0.002, Table 1).

#### 3.2.3. Association between TNF-*α* Serum Levels and BDI-II Scores

There was no statistically significant correlation between TNF-*α* levels and BDI-II scores at time point 1; however, we observed a statistically significant correlation at time point 2 (*r* = −0.682, *p* = 0.02).

### 3.3. Group-to-Group Differences

#### 3.3.1. TNF-*α* Serum Levels

There was no statistically significant difference in the levels of TNF-*α* between patients in the psychiatric day care unit and the psychotherapeutic unit at time point 1 (data not shown); at time point 2 TNF-*α* levels were significantly higher in the psychotherapeutic unit cohort than in the psychiatric day care unit cohort (*T* = −14.03, *p* < 0.001, Figure 1).

#### 3.3.2. BDI-II Scores

There was no statistically significant difference in BDI-II scores between the psychiatric day care unit cohort and the psychotherapeutic unit cohort neither at time point 1 nor at time point 2 (data not shown).

The influence of potential confounders—age, gender, BMI, and treatment with antidepressants and/or antipsychotics—was evaluated. Neither of these potential confounders did exert a significant influence on the presented results.

## 4. Discussion

The concept of neuroinflammation has been proposed as an explanatory model for the development of mood disorders. In this study, we investigated the interaction between the inflammatory response system and depression, taking advantage of the measurement of the levels of the proinflammatory cytokine TNF-*α* in two cohorts of depressed patients.

A meta-analysis by Dowlati and colleagues concluded that baseline TNF-*α* levels varied in a range of 0.18 to 77.68 pg/ml in depressed and 0.37 to 36.04 pg/ml in nondepressed cohorts [24]. The majority of authors found depressed patients to have 10–60% higher TNF-*α* levels compared to healthy controls [25–27]. There are only sparse and country-specific data showing slightly reduced TNF-*α* levels in depressed individuals [28, 29]. However, information about the duration of depressive symptoms before the measurement of TNF-*α* levels is absent in most publications.

In our study, depressed patients were divided into two cohorts: one cohort was treated at a psychiatric day care unit, whereas the other cohort was treated at an inpatient psychotherapeutic unit. Patients were investigated at two time points: shortly after admittance to the respective treatment and 4–6 weeks later.

According to the current evidence [24], we expected a decline of depressive symptoms to be accompanied by a reduction of TNF-*α* levels at time point 2, indicating a regression of (neuro)inflammation. However, in patients receiving CBASP in the psyotherapeutic unit, we observed a significant increase of TNF-*α* levels during the study period: at the end of the treatment, TNF-*α* levels were significantly higher than at the beginning and significantly correlated with reduced BDI-II scores at this time point. In the psychiatric day care unit cohort, there were no significant changes in TNF-*α* levels, and BDI-II scores were only slightly reduced. A possible explanation for the observed increase of TNF-*α* levels in our study instead of the expected decline is that the TNF-*α* increase might be compensatory since TNF-*α* is known to enhance cellular differentiation and proliferation [30, 31], thereby promoting neuroplasticity. Similar effects have also been reported for other neurotrophins, e.g., brain-derived neurotrophic factor (BDNF) [32]. This hypothesis is supported by evidence indicating that treatment with antidepressants may artificially reduce TNF-*α* levels. For example, Almishri and colleagues have reported that treatment with mirtazapine was associated with an attenuation of Concanavalin A-stimulated early innate immune responses in the liver, including inhibition of hepatic macrophage/monocyte activation and decreased production of hepatic macrophage/monocyte-derived proinflammatory cytokines (e.g., TNF-*α*) and chemokines [33]. Also, Li et al. have demonstrated that an eight-week treatment of depressed patients with venlafaxine may reduce TNF-*α* levels in treatment responders [34]. Similarly, eight-week treatment courses with sertraline [35] or fluoxetine [36] have been reported to be associated with reductions of TNF-*α* levels.

Yet, it must be stressed that multiple studies have examined the effects of antidepressants on TNF-*α* levels and, overall, the results remain inconclusive: psychopharmacological interventions have been associated with increased, decreased, or unaltered levels of TNF-*α* [27].

**Figure 2:**
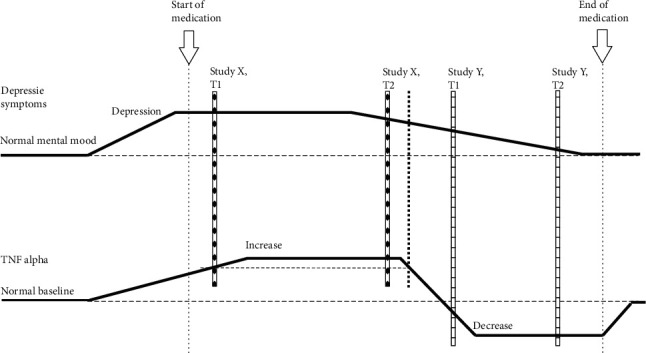
Potential time course of TNF-*α* levels with regard to the onset of depressive symptoms and medication. This hypothetical model could help to explain variations of TNF-*α* levels and contradictory findings in the literature as well as the differences observed between our two cohorts. As described in the discussion, TNF-*α* levels could slowly rise at the onset of depressive symptoms in order to compensate for neuronal disturbances and therefore show higher levels in this period compared to baseline. During recovery of depressed patients, TNF-*α* levels could decrease again and could even be reduced below baseline due to antidepressant medication. The different types of columns indicate two different potential studies (study X/oblique-striped columns vs. study Y/cross-striped columns) with two measurement time points each (T1 and T2). Our PTU-data would be best presented by the measurement time points indicated by study X. PDC-data of T2 would be located as indicated by the dotted line next to study X, T2. Y relates to other studies in the literature.

It is conceivable that variations of TNF-*α* levels and contradictory findings in the literature as well as the differences observed between our two cohorts might be explained by the time point of investigation. The majority of reports in the literature do not specify when exactly patients were investigated in relation to the occurrence of first depressive symptoms. As depicted in Figure 2, TNF-*α* levels could slowly rise at the onset of depressive symptoms in order to compensate for neuronal disturbances and therefore show higher levels in this period compared to baseline. During recovery of depressed patients, TNF-*α* levels could decrease again and could even be reduced below baseline due to antidepressant medication.

Therefore, a study such as ours which investigates patients relatively early after the onset of symptoms (indicated by X in Figure 2) (although only in tendency as it was difficult to determine the exact time point of onset) might find a situation as indicated by the oblique-striped columns in Figure 2: at time point 1 (T1) TNF-*α* levels might be higher than baseline but still on the rise, whereas depressive symptoms are already fully developed, as demonstrated by high BDI-II scores. Concerning the findings in the PTU TNF-*α* levels have reached their peak at time point 2 (T2), whereas depressive symptoms have already started waning. Values of the PDC would be best explained by the dotted line in this model. A different study (indicated by Y, cross-striped columns in Figure 2) might investigate patients who have already been suffering from longer periods of depression and who still show depressive symptoms (even though slightly reduced) but already exhibit strongly reduced TNF-*α* levels due to antidepressant medication. In such a study, it would be expected that at T2 depressive symptoms would have almost vanished. This hypothesis about the time course of TNF-*α* levels in depression could in general explain the inconsistency of TNF-*α* levels determined in different cohorts of depressed patients investigated thus far. On the other hand, the literature also shows some (though less pronounced) variation of TNF-*α* levels between healthy control groups. However, these comparatively small variations may be owing to technical differences in the measurement of TNF-*α*.

It must be emphasized that this model represents merely a basis for further discussion. Future studies on TNF-*α* levels in depressed patients should carefully consider and report the duration of depressive symptoms, even though, admittedly, the exact onset of a depressive episode is difficult to determine.

As with other publications on TNF-*α* levels in depression, limitations of our study mainly arise from the relatively small study cohort. In contrast to the psychiatric day care unit, the psychotherapeutic unit in our study is an inpatient facility. This may have influenced the results, for example, due to psychosocial components. Furthermore, there were no reliable data available with regard to the exact phase of illness before treatment or with regard to possible pretreatments. However, usually only little time has passed until admission and as mentioned above, this may be limitations that also affect other studies. Due to the design of the study and, more importantly, for ethical reasons, a randomization of the patients was not performed. Moreover, there was no dedicated sample size estimation but a naturalistic cohort recruitment.

## 5. Conclusions

In conclusion, in order to be able to compare different cohorts with regard to cytokine levels, it is of utmost importance to ask patients when first depressive symptoms emerged and to thoroughly document the further psychopathological development as well as the time course of medication. As a future perspective, it would be of crucial interest to investigate the time course of TNF-*α* levels in more detail with more frequent measurements.

Despite the limitations of this study, the correlation of BDI-II scores and TNF-*α* levels as well as the elevation of TNF-*α* levels at T2 in the psychotherapeutic unit cohort was a statistically robust finding. It may be speculated that the highly specific form of psychotherapy for depression applied in this study, i.e., CBASP, may exert a particular effect on neuroinflammation, especially on the levels of TNF-*α*. To the best of our knowledge, this is the first study that demonstrated an influence of CBASP on TNF-*α* levels in depressed patients. Future studies are warranted to confirm this observation.

## Figures and Tables

**Table 1:** Patient characteristics as well as BDI-II scores and TNF-*α* levels at time points 1 and 2 of both cohorts.

	Psychiatric day care unit (*n* = 16)		Psychotherapeutic unit (*n* = 18)	
	Mean value	Standard deviation	Mean value	Standard deviation
Age (years)	43.38	14.01	36.89	10.58
Gender (m/f)	4/12	—	4/14	—
Moderate depressive episode	2	—	2	—
Severe depressive episode without psychotic symptoms	5	—	2	—
Recurrent depressive disorder, current episode moderate	5	—	9	—
Recurrent depressive disorder, current episode severe without psychotic symptoms	4	—	5	—
BMI (body mass index)	27.32	6.02	24.11	3.12
Treatment with antidepressants (y/n)	13/3	—	11/7	—
Treatment with antipsychotics (y/n)	1/16	—	0/18	
Length of treatment (days)	45.86	17.63	60.35	13.33
BDI-II_T1 (score)	29.69	10.22	29.17	8.85
BDI-II_T2 (score)	22.60	8.98	15.94	13.41
TNF-*α*_T1 (pg/ml)	6.77	4.47	6.66	2.75
TNF-*α*_T2 (pg/ml)	6.57	4.87	50.39	11.60

## Data Availability

The availability of the underlying data related to the submission is given.
